# Supplementary material for: Cross-feeding affects the target of resistance evolution to an antifungal drug
Source: PLoS Genet. 2023 Oct 19;19(10):e1011002. doi: 10.1371/journal.pgen.1011002 (PMC10617708; doi:10.1371/journal.pgen.1011002)
Supplement: Attachment — Submitted filename: Reviewer response.docx [file pgen.1011002.s015.docx]

## Reviewer #1

This study was designed to comprehensively elucidate the routes towards in vitro resistance to antifungal drug 5-fluorocytosine (5FC) in two different wild and prototrophic strains of S. cerevisiae. Selection for resistant mutants was done by starting with a preculture grown in YPD and followed by selection and enrichment for mutants in a 96-well format using SD medium supplemented with 1.56 μg/mL 5-FC. This procedure produced 682 5-FC-resistant mutants in the two strain backgrounds, about 1/3 of which were petite (non-respiring) and the rest had functional mitochondria. The authors showed that the petite 5-FC resistant mutants had also acquired resistance to fluconazole, consistent with extensive literature on the subject, and perhaps showed improved fitness in the echinocandin micafungin, although that was more difficult to conclude due to confounding growth effects. The vast majority of non-petite 5-FC resistant mutants had mutations in FUR1, which is in contrast to clinical mutations leading to 5-FC resistance in fungal pathogens, which encompass mutations in FUR1, FCY1, and FCY2 and also unexpected given the fact that the fcy1∆ mutant is highly resistant to 5-FC in their experimental system. The authors investigate this discrepancy and conclude that it’s due to the design of the in vitro evolution experiment, where any arising fur1 mutations would produce 5-fluorouracil that would be toxic to neighboring cells, including both wild type and fcy1 mutants, but not to the fur1 mutants themselves. They provide evidence for this conclusion by showing that in the medium preconditioned with a fur1 mutant but not with a wild type strain, fcy1 mutant no longer has a fitness advantage in the presence of 5FC. The major strengths of the study are the use of two different protrophic strains of S. cerevisiae and a clear description of the study design and its outcomes.

### Major comments

The major weakness, of which the authors appear to be aware, is that the in vitro evolution experiment produced a very narrow set of outcomes that do not correspond to those described in the clinical setting. The latter is not a weakness per se, as in vitro evolution experiments conducted in a model yeast are not expected to fully recapitulate the mutational signatures occurring in fungal pathogens occupying their host niches. However, in this particular case, any potential advantages of using S. cerevisiae for experimental evolution did not come to bear either, as the experimental design appeared to strongly select for mutations in FUR1 only. Given this outcome and the followup experiments, the two novel conclusions/hypotheses that can be drawn from this study are: 1. Petite strains with increased drug efflux may have increased fitness in 5-FC, which may be relevant clinically, and 2. Fungal cell density can affect the paths of evolution of antifungal drug resistance, e.g., via crossfeeding between fungal cells. Both hypotheses are potentially interesting for the field of antifungal drug resistance, but neither is explored very much. My suggestion for the authors, in order to make this study more convincing, thorough, and appropriate for PLoS Genetics, is to test the 2nd hypothesis by altering the setup of experimental evolution and showing that fcy1 mutations can now be obtained, e.g., at lower cell densities or when the strains carry a 2nd copy of FUR1. In this case, true fluctuation experiments (see comment below) can be useful to measure mutation rates and identify the genes responsible at different cell densities or when 2 copies of FUR1 are present. These experiments do not need to be done at large scale or necessitate additional costly WGS. Given the narrow range of resistant mutants, Sanger sequencing of candidate genes should be sufficient.

We appreciate Reviewer 1’s constructive evaluation and took into account the recommendations to strengthen the study. In general, we decided to shift the focus of the study to *rho*^+^ mutants, for which we were able to explain the resistance mechanism in more detail. Out of two main genes involved in 5-FC resistance, we find our experimental setup does strongly select for mutations in *FUR1*, but we also bring evidence to explain why it selects against mutations in *FCY1*. To further demonstrate that fungal density affects the paths of resistance evolution in this context, we performed a competition experiment using our two null mutants of *FCY1* and *FUR1*. We did not repeat the evolution experiment at different densities because it would be very unlikely to find mutants at low density given that mutation rate is too low. Indeed, previous attempts of cultivating the strains in the absence of selective pressure had not allowed to generate resistant mutants (see lines 212-213 and 712-715 in the first submission). We considered the possibility of using a mutagenic agent to increase the mutation rate but ultimately, the experiment would not have yielded enough mutants (many of which we expect would have been LOF *fur1* mutants). In the end, we focused on what we consider is the most important aspect of the hypothesis: when LOF *fcy1* and *fur1* mutants have arisen in the population, what is their outcome? In particular, does cell density and cross-feeding favor the selection of one type of mutant over the other? To verify that, we grew both null mutants in the absence of selective pressure, mixed the cultures in equal volumes and inoculated several dilutions on large petri dishes with 5-FC. Mutants were streaked to confirm they were resistant, and tested by multiplexed colony PCR to identify them. A Chi-square test of independence confirmed that there is a significant relationship between the dilution and the counts of *fcy1*Δ/*fur1*Δ mutants. Specifically, inoculating at 10^-3^ OD resulted in a high density of colonies, with all tested colonies identified as *fur1*Δ mutants. On the other hand, inoculating at 10^-5^ OD resulted in a low density of colonies, half of which were identified as *fcy1*Δ mutants.

To further validate the cross-inhibition effect, we spotted the *fur1*Δ mutant on a lawn of *fcy1*Δ mutant, which resulted in a very clear inhibition zone, supporting the link between density and 5-FU cross-feeding. The new results are now featured in Fig 6CD, with uncropped pictures of the inhibition assay plates in Supp Fig 8.

### Minor comments

1. The authors use the term “fluctuation assay” inappropriately. What they did was select for resistant mutations in the presence of 5-FC in liquid, whereas fluctuation experiments are done in drug-free liquid cultures, resulting in “fluctuating” numbers of spontaneous mutants that occur without selection and that are then detected as varying numbers of colonies on solid drug-containing medium.

The term “fluctuation assay” has been changed to “evolution experiment”.

2. How distantly related are the two S. cerevisiae strains?

The two strains have 0.39% nucleotide divergence (averaged across chromosomes). This is roughly what is expected for within-species diversity in many fungi. This is now mentioned in the methods section.

3. In figure 3A, the asterisks indicate statistical significance; however, in some cases it is not clear what the differences between petite and non-petite strains are. For example, for LL13-040 in caspofungin, there are 4 asterisks, but it’s not clear whether rho+ or rho- strains have better fitness. Another way of showing the data is necessary to make that clear.

*rho*^+^ and *rho*^-^ mutants are now displayed side by side in boxplot format with data points in Figs 2 and 3A. We initially used only the highest concentration of each antifungal (out of 3) to calculate the relative fitness. However, as a result, the dynamic range for antifungals other than 5-FC was quite small, resulting in a lot of noise when comparing relative fitness values. We decided to set a cutoff for the AUC value (absolute fitness) at 6,000 arbitrary units. In other words, we selected the drug concentrations which allowed sufficient growth, as indicated by the mean AUC obtained for each background (across all mutants) in each condition. If this mean AUC was above 6,000 a.u. for at least one background, we excluded this drug concentration and selected a lower concentration (if it met the criteria). In this version of the manuscript, we now calculate the fitness values in caspofungin, micafungin and nystatin with the lowest concentration tested: caspofungin, 0.25 μg/mL; micafungin, 0.0625 μg/mL; nystatin, 4 μg/mL. Concentrations for fluconazole, 5-FC and 5-FU stay unchanged.

4. In figure 4C, I recommend replacing FCY1 and FCY2 with fcy1∆ and fcy2∆, as they are shown in 4D.

Figure 4C now features the recommended notation.

5. Sentence "All identified mutations in these three genes (n=7) are predicted to impact conserved residues with five out of seven also predicted to impact protein stability" (line 333) has not supporting evidence.

The methods section contained the related paragraph “Predictions of impact of mutations” to specify that the predictions were obtained using mutfunc (PMID: [30573687](https://pubmed.ncbi.nlm.nih.gov/30573687)). To improve clarity, the sentence now also mentions mutfunc.

6. Sentence “…with all Sanger sequenced samples confirming what had been detected by WGS (Fig 5A)” (line 356) conflicts with what is actually shown in 5A: based on the upset plot, it appears that a few mutations detected by samtools and gatk were not detected by Sanger sequencing.

The sentence is correct. The issue is related to the fact that not all WGS samples have been sequenced by Sanger (because of a lack of DNA material). The explanation was present at lines 851-852 in the first submission. To add more details, the sentence now features the number of samples sequenced by Sanger (268 out of 282 samples sequenced by WGS). To be clear, the 268 samples also include our 6 controls (WT, *fcy1*Δ and *fcy2*Δ for both backgrounds), which we confirmed by Sanger to be carrying the WT allele of *FUR1*.

## Reviewer #2

This study investigates the spectrum of spontaneous mutations conferring resistance to flucytosine (5-FC) in two different wild haploid isolates of S. cerevisiae. In both isolates, FUR1 deficiencies constitute the majority of the 5-FC resistant derivatives. Deficiencies of FCY1 were not obtained due to the manner in which the screen was implemented. Interestingly, while fcy1∆ mutants were resistant to 5-FC in isolation they were not resistant when co-cultivated together with wild-type, which apparently cross-feed the 5-FU derivative of 5-FC and therefore intoxicate the FCY1-deficient cells. Another interesting finding was the frequent isolation of petite mutants that exhibit 5-FC resistance in spite of their fitness defects in glucose medium. Such mitochondrial deficiencies are known to activate the Pdr3 transcription factor, which induces expression of several ABC transporters and other factors that promote efflux of diverse small molecules. Indeed, the petite mutants were shown to confer decreased retention of a fluorescent dye (i.e. elevated efflux) and also cross resistance to fluconazole (transported by Pdr5, a target of Pdr3). However, there were no follow up experiments demonstrating direct involvement of Pdr3 and Pdr5 in 5-FC resistance of petite mutants. Surprisingly, the mitochondrial deficiencies generally conferred strong cross resistance to Micafungin but not Caspofungin. These echinocandins are important antifungals in clinical settings, so it would be interesting to determine with direct experiments whether Pdr3 is somehow conferring Micafungin resistance in the petite mutants. The manuscript is framed around evolution of drug resistance in clinical settings, but the authors should acknowledge that the findings of this study apply mainly to haploid pathogens and not diploids such as Candida albicans. In summary, the study was interesting, well executed, and well written though a few additional experiments involving Pdr3 would strengthen the final conclusions. Major and minor suggestions for improvement are listed below.

### Major comments

The “predicted alternative path to resistance” involving mitochondria, Pdr3, and Pdr5 that is illustrated in Fig. 1 is very interesting and novel, but not experimentally tested in this study. Do pdr3∆ mutations diminish resistance to 5-FC (and 5-FU) in petite cells as predicted? What about pdr5∆ mutants or any other suspected transporters that regulated by Pdr3?

We thank Reviewer 2 for their comments and suggestions. We did not explore this aspect much further in the initial submission because we focused on the *rho*^+^ mutants, which we thought were more relevant to resistance mechanisms that could be encountered in clinical settings. Upon reception of the reviewers’ recommendations, we did undertake several experiments to try and bring new evidence that loss of mitochondrial function is related to the upregulated efflux of 5-FC. Unfortunately, we hit several roadblocks, which ultimately did not bring new results and prompted us to shift the focus of the study to *rho*^+^ mutants. First, we tried to investigate the role of key transcription factors: paralog pairs *PDR1/PDR3* and *PDR8/YRR1*, as well as their putative target, efflux pumps -encoding *PDR5* and *FLR1*, respectively. Since these transporters belong to distinct classes (ABC and MFS, respectively), we expected to observe interesting phenotypes. However, because loss of mitochondria causes an alleviation of repression, we could not simply transform the *rho*^-^ mutants with plasmids expressing the genes (such as *PDR3*) under their native promoter. Instead, we decided to induce their expression from a galactose-inducible promoter in the laboratory strain BY4741. Results were difficult to interpret, which prompted us to switch to a design with an estradiol-inducible promoter. Unfortunately, the results still could not be reliably interpreted. Notably, the phenotypes varied drastically if the induction was done in the precultures. There was no apparent difference between the constructions, even in the absence of estradiol, in the presence of 5-FC +/- estradiol or fluconazole +/- estradiol. Finally, the construction carrying a control vector was often growing in conditions where we would expect growth to be inhibited. Ultimately, because the design involved the use of a uracil auxotrophic marker, it is possible that overexpressing a transporter which was potentially pumping out uracil was interfering with the selection of the constructed strains. Changing the design to G418 selectable constructions might have yielded better results but ended up being too much work and additional cost, and would have delayed further the resubmission of our manuscript. We will therefore leave the results relating to this part as is, providing a starting point for future investigation.

### Minor comments

1. The study focuses on the evolutionary trajectory of 5-FC resistance in a haploid model organism that is certainly relevant to C. glabrata and other haploid pathogens. But in what ways is this study relevant and not relevant to diploid pathogens that possibly cannot lose mitochondrial function?

Loss of mitochondrial function is likely not a viable evolution path in diploid species. It does however shed light on the poorly characterized efflux-mediated resistance to 5-FC. Future work is needed to elucidate the molecular mechanisms at play here, but one can speculate they do not necessarily involve loss of mitochondrial function. Instead, a single point mutation in the promoter of a transcription factor regulating the expression of one or several efflux pumps is likely enough to confer resistance. This has been shown in several contexts, including (as pointed out by Reviewer 2) in clinical isolates of haploid pathogens (PMID: [16569856](https://pubmed.ncbi.nlm.nih.gov/16569856)), but also in diploid pathogens, where homozygous resistance-conferring mutations are selected through loss of heterozygosity events (PMIDs: [26721282](https://pubmed.ncbi.nlm.nih.gov/26721282/), [22924823](https://pubmed.ncbi.nlm.nih.gov/22924823/)).

Regarding *FUR1*-mediated resistance, it has been shown that natural resistance to 5-FC in *Candida albicans* is specific to clade I strains and is caused by a single nucleotide substitution resulting in a R101C mutation (PMID: [15155225](https://pubmed.ncbi.nlm.nih.gov/15155225)). What is particularly interesting is that heterozygous strains show reduced susceptibility to 5-FC (whereas homozygous strains are resistant). Although we have not detected this specific mutation in our dataset, our complementation assays (see below) reveal other mutations display a similar phenotype.

2. Cross resistance of petite mutants to fluconazole and micafungin is interesting and relevant to the overall impact of the manuscript. Does Pdr3 contribute to this phenomenon?

We agree these are interesting avenues of research. However, exploring the underlying molecular mechanisms would be out of the scope of this study. The observed correlation between rhodamine efflux and fitness in fluconazole is a good indication that efflux is most likely involved, which could explain cross-resistance. *PDR3* could be involved but since it has a paralog, *PDR1*, the experiments would have needed to show the role of *PDR3*, while ruling out the role of *PDR1*, which would have required extensive work.

3. The analyses of 5-FC resistant isolates that were not petite is confusing. 258 of 276 sequenced genomes contained a mutation in FUR1, but the calls were extremely noisy with over 100 additional mutations in each genome and inconsistency between the calling methods. Sanger sequencing of FUR1 was performed on a large portion of them, but the supplemental tables and the descriptions in the Results do not make it clear which isolate has which mutation (and what sources of evidence support or contradict it). Can a new supplemental table be generated that lists each isolate plus the Sanger sequencing and whole-genome sequencing outcomes (samtools and gatk methods)?

We agree that the WGS analysis is noisy. This results from the set of filters used to make sure that there were no mutations in other well-known target genes such as *FCY1* or *FCY2*. In the end, we felt the noise was not such an issue as long as we could identify the most likely cause of resistance. Unfortunately, too many WGS studies report either a very low noise (which likely means the criteria for SNP filtering were very stringent, leaving some true positives undetected), or attribute the resistance phenotype to a mutation without much evidence to support it. Our complementation assay (see point below) confirms the most likely cause of the resistance phenotype is indeed the identified LOF mutations in *FUR1*.

Regarding the list of mutations, Supplemental Data 1 (submitted with the first version of the manuscript) contains all relevant data. Its most recent version (Supp Data 2) now comes with a more explicit caption (previously below the table, now in the main text as well as above the table) and features the genetic background and if the strain was used in the validation and/or complementation assay.

Additionally, we are now providing a table (Supp Data 3) where each row features the relative fitness in YPD, SD, SD + 5-FC and SD + 5-FU (at 30°C) for strains with a detected mutation in a gene likely to cause resistance. We believe this format of reporting will help the community highlight the most prevalent resistance-conferring mutations.

4. The analytical pipeline did not make use of complementation testing, which is gold-standard for identification of causative mutations.

We thank Reviewer 2 for their helpful suggestion. We now report the results of a complementation assay (Fig 5B), in which we used 32 strains transformed with either a control vector (pMoBY) or the same vector expressing *FUR1* from its native promoter (pMoBY-*FUR1*). For all strains in which a mutation introducing a stop codon was detected, expressing *FUR1* restored sensitivity to 5-FC (whereas the presence of the control vector did not). We interpreted these results as strong evidence that resistance is caused by the detected mutation which imparts *FUR1* loss of function. Interestingly, some resistance mutations could not be masked by the WT copy of *FUR1*, but we find the corresponding residues to be located at the interface between two Fur1 monomers (Fig 5C). There is a good chance the observed dominant negative effect results from misassembly of the heteromer formed by the mutant allele and the WT allele. If confirmed (which would be out of the scope of this study), this observation would corroborate previous reports of the resistance-conferring heterozygous mutation R101C in *C. albicans* (PMID: [15504867](https://pubmed.ncbi.nlm.nih.gov/15504867)). Similarly, the residue is thought to be involved in the interface between two monomers.

In a few places, complementation could resolve and clarify the findings. For example, four resistant isolates did not have mutations in FUR1 and yet behaved like fur1∆ mutants (line 338). Are these mutants complemented by FUR1 on a plasmid, or non-complemented by mating to a fur1∆ strain? What about some of the other ones where the methods were inconsistent or the evidence was incomplete? This data could be added to the new supplemental table proposed above with the overall goals of determining the genetic cause of resistance in each isolate and evaluating the consistency of the methods.

We have specifically chosen to carry the complementation assay with most validation mutants for consistency. I believe the reason why no mutation could be detected in the four mentioned isolates is simply because of low coverage (at least for 3 of them). Also, we wanted to make sure we could compare the results of the complementation assay with the nature of the detected mutation. Typically, we expect strains carrying an allele encoding for a truncated Fur1 to behave like the null mutant, which is what we observe. We also observe comparable phenotypes for strains carrying the same mutation, even though they evolved from two distinct parental strains. This further confirms resistance is caused by the detected mutation.

## Reviewer #3

### Major comments

I am missing data on the clinical relevance of this study. It would be interesting and important to screen S. cerevisiae clinical isolate for resistance to 5-FC and screen for the mutations identified here (or perhaps novel ones).

*S. cerevisiae* is rarely a pathogen, but some strains have been shown to cause infection, with some severe fungemia cases reported (PMIDs: [16267727](https://academic.oup.com/cid/article/41/11/1559/356155), [26779173](https://pubmed.ncbi.nlm.nih.gov/26779173), [29132794](https://www-sciencedirect-com.acces.bibl.ulaval.ca/science/article/pii/S1156523317301087)). It is also used as a model for pathogens in many contexts, including to study drug resistance (PMIDs: [15371350](https://pubmed.ncbi.nlm.nih.gov/15371350), [23593013](https://pubmed.ncbi.nlm.nih.gov/23593013), [36173330](https://pubmed.ncbi.nlm.nih.gov/36173330) to cite a few). It is an interesting model from a technical viewpoint as well, since many tools for genetic engineering are available and it is amenable to high-scale experiments. Despite the use of non-pathogenic strains in our study, we do observe clinically relevant mutations conferring resistance to a commonly used and essential antifungal. For example, the mutation G190D identified in *FUR1* in our dataset was previously shown to confer resistance to 5-FC in clinical isolates of *Nakaseomyces glabrata* (PMID: [20038613](https://pubmed.ncbi.nlm.nih.gov/20038613)).

I am curious how the authors decided on which antifungal drug concentrations to use. Was this based on some published work? Random? Subinhibitory versus high concentration (in the case of fluconazole)?

We performed several preliminary tests on petri dishes with the same minimal medium to evaluate which concentration would be enough to observe a difference between the WT and some of the generated mutants. We selected 3 concentrations which were all tested in our automated growth measurements. These concentrations are in the range used for the corresponding molecules in similar conditions in other studies. The choice of the final concentration used to calculate the fitness values is explained in more detail in the response to Reviewer 1.

Because this manuscript has a ton of data/results, it would be very helpful to have some sort of introduction to both, results and discussion, so the reader can be brought up to speed quickly.

We agree there are a lot of results, but we believe the results and discussion sections are appropriately introduced. However, we did notice that confusion could arise whenever comparing the experiments as they are described in the results section to their corresponding description in the methods section. To address this, we reorganized the methods section to better reflect the workflow (see response below). Two tables which were previously in the main text were also moved to Supplemental Data, hopefully leaving the main text less crowded.

Overall, there needs to be a better description of all the different assays. It is difficult to keep track of all of them. I think some sort of flow chart might work. Alternatively, or in conjuncture, start each method part with a sentence that states the purpose of the experiment might also help.

The methods section was significantly rearranged to improve clarity. A short description of the purpose of the experiment(s) now precedes each detailed protocol.

It is not clear from the methods, which strains were used for the fluctuation analyses? This needs to be clarified. The same goes for parts of the methods.

Haploid strains LL13-040 and NC-02 (both mat **a**) were used as the parental strains in the now renamed “evolution experiment”. Details have been added to the corresponding methods section.

Fluctuation analyses usually give a rate of (in this case resistance to drug?). I am missing this here.

This, and the issue brought up above, appear to be related to the misuse of the term ‘fluctuation’, which has since been changed to ‘evolution experiment’.

I am not clear about the reason of generating haploid progeny. When and for which experiment were they used?

As mentioned in the methods, the use of haploids allows the identification of recessive mutations. The pathogen with the shortest evolutionary distance to *S. cerevisiae* is a haploid *N. glabrata*. In general, we also use this model to facilitate genetic constructions whenever the context allows it.

The authors identified cross-resistance of rho- mutants to 5-FC and fluconazole and tested efflux capacity. I am guessing that the reason no genomic analysis was done was due to unsuccessful DNA extraction with the method employed? I think genomic data for these mutants would add important information and make this a stronger manuscript. For example, they could look at their genome sequence for mutations in genes known to impact resistance to fluconazole and/or test the expression of genes known to affect fluconazole resistance?

As Reviewer 1 pointed out, generating new WGS results would have been too costly in terms of time and effort given that the focus of the manuscript is on the other findings. We agree this is an interesting avenue of research, but it would also be out of the scope of this study, in which we decided to focus on *FUR1* mutations. Specifically, in this revised version of the manuscript, we bring new evidence that loss of function in *FUR1* and 5-FU cross-feeding are key to directing resistance evolution.

It would be great to have a figure with the FUR1 ORF and the locations of all unique mutations.

The suggested figure was added as Supp Fig 5.

Along this line, did the authors introduce a few of the major loss of function mutations into the parental strains to confirm the resistance phenotype?

The identified *FUR1* mutations were not recreated in the parental strains, only the complete deletions were constructed. However, we did confirm that 5-FC sensitivity is restored upon expression of *FUR1* on a plasmid in most of the 32 tested mutants (see response to Reviewer 2 for more detail).

Also, in the text it says ‘Supplemental Data 1” for the list of SNPs/indels but they were submitted as suppl 2 excel file, which was a bit confusing.

I apologize for the confusion, the submission platform apparently renamed all supporting files, something that should have caught my attention. I will make sure that the appropriate numbering is kept throughout in this version and the subsequent ones.

I am curious if the authors have thought about trying a different selective marker for their fluctuation analysis (Nat or Hyg) and then screen the colonies for 5-FC resistance. Would that have perhaps yielded some Fcy1 mutants?

One background is marked with Nat, the other one with Hyg. We do observe parallel evolution of resistance, which leads us to believe that using the other combination of markers would not have impacted the end result.

Discussion, starting at line 479: Can the authors please speculate on what type of unknown mechanism could be responsible and how they would test this? Drug efflux is quite general. In addition, other points made in this part are mostly speculation and I would shorten it a bit.

This section of the discussion was rewritten to better reflect what was observed in our study, and how it corroborates reports in the literature. In summary, we believe resistance to 5-FC and fluconazole could be caused by two mechanisms, with overlapping regulatory pathways, although confirming this hypothesis would require future investigation.

### Minor comments

Title of figure 1? Legend is overly long and some details are redundant to what’s described in-text.
Figure S2: I am not sure this figure is necessary.
Line 761: Maybe a reference to Figure S6 would be helpful to visually see what the 96 well plates look like.
Line 779: Please define QC (I am assuming it’s Quality Control?)
Line: 787: which WT strain?
Line 795: what type of centrifuge? 230 g can be all kinds of speed depending on the exact machine.
Figure S3 legend: the sentence ‘A dashed gray line….’ Needs some better wording.
Line 814: Please define SSC and FCS. Not everybody is familiar with flow cytometry
Lines 890-901 should be written in sentence format.
Lines 920-931: This methods may be better positioned before ‘DNA extraction and sequencing.

We appreciate Reviewer 3’s attention to detail, however we respectfully disagree with some of the suggestions. We believe more information is better and leave the ultimate decision to the editor. On the other hand, we don’t believe adding a depiction of a 96-well plate would prove relevant. 230 g can indeed correspond to different speeds depending on the centrifuge, which is why a measure of acceleration (g), which constitutes a universal parameter, is provided.

The following modifications have been made as suggested:

- QC now reads “quality control”,
- the “WT” strain now reads “the parental strain” (which is adequately described in the first methods section),
- “dashed gray line” now reads “gray dashed line”,
- We now mention “proxy for cell size” and “proxy for cell granularity” for the FSC and SSC values, respectively,
- WGS data filtering criteria are now listed in sentence format,
- The methods section was rearranged as suggested.
